# Editorial: Imaging in Ophthalmology

**DOI:** 10.3390/jcm11185433

**Published:** 2022-09-15

**Authors:** Mariantonia Ferrara, Yalin Zheng, Vito Romano

**Affiliations:** 1 Manchester Royal Eye Hospital, Oxford Road, Manchester M13 9WL, UK; 2 Department of Eye and Vision Science, University of Liverpool, Liverpool L69 3BX, UK; 3 St Paul’s Eye Unit, Royal Liverpool University Hospital, Liverpool L69 3BX, UK; 4 Eye Clinic, Department of Medical and Surgical Specialties, Radiological Sciences, and Public Health, University of Brescia, 25121 Brescia, Italy; 5 ASST Civil Hospital of Brescia, 25123 Brescia, Italy

Over the last decade, ophthalmology has significantly benefited from advances in vivo non-invasive ophthalmic imaging techniques that play currently a fundamental role in the clinical assessment, diagnosis, management, and monitoring of a wide variety of conditions involving both the anterior and posterior segment [1,2,3,4,5,6]. Imaging technologies, including anterior and posterior segment optical coherence tomography (OCT), OCT angiography, wide-field retinal imaging, specular and confocal microscopy, corneal topography, and ocular ultrasound, have dramatically improved the morphological and functional evaluation of ocular structures, both in healthy and pathological eyes [1,2,3,4,5,6,7,8,9,10,11]. 

The detection of tissue microstructural changes, even at the subclinical level, can improve our ability to not only make an appropriate diagnosis, but also to elucidate pathogenetic mechanisms and to plan an appropriate management strategy for several pathological conditions. In this regard, for instance, an analysis of the retinal and corneal changes associated with SO tamponade provided important information on the potential effects of this compound on ocular tissues and facilitated the early detection of complications [12,13,14,15]. This aspect is of great clinical relevance, especially when considering that SO-related complications can be severe and potentially sight-threatening [16]. Furthermore, imaging techniques allow the identification of new biomarkers with different potential applications, including the detection and prediction of progression or responses to the treatment of common ocular diseases (e.g., age-related macular degeneration, AMD, diabetic retinopathy, DR, and myopic choroidal neovascularization) [17,18,19]; the early detection of systemic diseases, including hypertension [20] and multiple sclerosis [21]; the prediction of functional outcomes after surgical procedures [22]; or the detection of potential complications associated with systemic dugs [23].

With regard to the anterior segment, corneal topography and tomography have an established role in the accurate evaluation of the corneal shape as well as in the preoperative assessment for refractive and cataract surgery [24]. They are also relevant in the diagnosis, surgical planning, and long-term monitoring of various corneal pathologies, including keratoconus [25,26,27], ectatic corneal diseases, and pterygium or corneal scars [28,29]. It worth noting that keratometry measurements may significantly differ on the basis of the methodology used (e.g., anterior segment-OCT vs. Pentacam) [30]. Specular microscopy is a fundamental tool in the assessment of corneal and diagnosis and in the management of corneal endothelial disorders [31]. This technique can be also used to assess corneas stored in cold storage or in organoculture using an active storage machine [32]. Confocal microscopy allows for the detailed analysis of corneal nerves as well as for understanding their important role in the corneal structure and function in common corneal diseases such as keratoconus [33] but also as early markers of ocular involvement in systemic diseases, such as type 2 diabetes [34].

With regard to posterior segment, the advent of OCT and OCTA and their recent developments has dramatically improved the assessment of retinal and choroidal disorders. The diagnosis and the management of medical retinal diseases, including AMD, DR, and retinal vein occlusion, has been optimized by the use of these techniques, and the need for more invasive investigations, such as fluorescein angiography, has decreased [35,36,37]. This shift has also been seen in the anterior segment [38,39,40,41,42]. The evaluation and management of vitreoretinal interface diseases have particularly benefited from these imaging techniques, which allow for detailed structural analysis of the retinal tissues and the identification of multiple anatomical findings for classification [43,44], differential diagnosis [45,46,47], surgical planning [48,49,50], prognosis [45,49,51], and long-term monitoring [50,52]. It has been recently suggested that retromode imaging modalities, which rely on confocal scanning laser ophthalmoscopic technology, may be a promising additional tool for the assessment of ERMs [53].

The possibility of combining different imaging modalities can optimize the processes of differential diagnosis, particularly in diseases sharing multiple common clinical aspects, including macular oedema of different etiologies [54] or inflammatory pathologies [55,56,57], as shown for chorioretinal lesions associated with Mycobacterium (M.) chimaera, M. tuberculosis, and other ocular granulomatous infectious diseases [58].

Finally, there is a growing interest in the use of artificial intelligence (AI) and deep learning in ophthalmology due to the promising results achieved in the detection of common ocular diseases such as AMD, diabetic retinopathy, and glaucoma, and the potential applications for screening, diagnosis, and monitoring of these conditions [59]. The high accuracy of a computer-aided diagnosis algorithm using deep convolutional neural networks in recognizing and classifying high levels of myopia through fundus images has also been reported [60]. Interestingly, an AI system based on transfer learning and deep learning has been successfully applied for meibography analysis [61].

In this issue, we aimed to highlight the multiple potential applications of imaging techniques in ophthalmology, and we hope that this will be appreciated by readers.
